# Characterization and comparative sequence analyzes of GABA receptor gene in Asian main malaria mosquito, *Anopheles stephensi*

**DOI:** 10.1186/1475-2875-9-S2-O15

**Published:** 2010-10-20

**Authors:** Saber Gholizadeh, Ameneh Karimi, Nooshin Bayat, Navid Dinparast Djadid, Sedigheh Zakeri

**Affiliations:** 1 Insectarium, Malaria and Vector Research Group (MVRG), Biotechnology Research Center (BRC), Pasteur Institute of Iran (PII), Pasteur Avenue, PO BOX 1316943551, Tehran, Iran

## Background

The mutations occurring in receptor genes of Gama Amino Butyric Acid (GABA) play important roles in resistance to cychlodien insecticides in mosquitoes. Here we report the sequence analysis of the *Rdl* gene from Asian main malaria mosquito, *Anopheles stephensi,* using specific primers in the polymerase chain reaction.

## Method and results

Mosquitoes were collected from Fars, Hormozgan, Kerman and Sistan & Baluchistan provinces of Iran. A 256bp sequence of GABA receptor showed 99-100% similarity between *An. stephensi* populations in Iran. Gas chromatography count was 41.63% with 86 amino acid sequences. Alteration in amino acid sequence that has been found in *An. stephensi* populations will be discussed in detail, in comparison with *An. gambiae.*

## Conclusion

Understanding of molecular structure of resistance can provide baseline data to evaluate population genetics of pesticide resistance, which will be applicable in operational vector control measures and sterile insect technique.
